# Developing a Bayesian hierarchical model for a prospective individual patient data meta-analysis with continuous monitoring

**DOI:** 10.1186/s12874-022-01813-4

**Published:** 2023-01-25

**Authors:** Danni Wu, Keith S. Goldfeld, Eva Petkova

**Affiliations:** 1 Department of Population Health, New York University Grossman School of Medicine, New York, USA; 2 Department of Child and Adolescent Psychiatry, New York University Grossman School of Medicine, New York, USA; 3 Nathan Kline Institute for Psychiatric Research, Orangeburg, USA

**Keywords:** Bayesian hierarchical models, Bayesian adaptive trial design, Bayesian simulation, International consortium for data sharing, Prospective individual patient data meta-analysis, COVID-19

## Abstract

**Background:**

Numerous clinical trials have been initiated to find effective treatments for COVID-19. These trials have often been initiated in regions where the pandemic has already peaked. Consequently, achieving full enrollment in a single trial might require additional COVID-19 surges in the same location over several years. This has inspired us to pool individual patient data (IPD) from ongoing, paused, prematurely-terminated, or completed randomized controlled trials (RCTs) in real-time, to find an effective treatment as quickly as possible in light of the pandemic crisis. However, pooling across trials introduces enormous uncertainties in study design (e.g., the number of RCTs and sample sizes might be unknown in advance). We sought to develop a versatile treatment efficacy assessment model that accounts for these uncertainties while allowing for continuous monitoring throughout the study using Bayesian monitoring techniques.

**Methods:**

We provide a detailed look at the challenges and solutions for model development, describing the process that used extensive simulations to enable us to finalize the analysis plan. This includes establishing prior distribution assumptions, assessing and improving model convergence under different study composition scenarios, and assessing whether we can extend the model to accommodate multi-site RCTs and evaluate heterogeneous treatment effects. In addition, we recognized that we would need to assess our model for goodness-of-fit, so we explored an approach that used posterior predictive checking. Lastly, given the urgency of the research in the context of evolving pandemic, we were committed to frequent monitoring of the data to assess efficacy, and we set Bayesian monitoring rules calibrated for type 1 error rate and power.

**Results:**

The primary outcome is an 11-point ordinal scale. We present the operating characteristics of the proposed cumulative proportional odds model for estimating treatment effectiveness. The model can estimate the treatment’s effect under enormous uncertainties in study design. We investigate to what degree the proportional odds assumption has to be violated to render the model inaccurate. We demonstrate the flexibility of a Bayesian monitoring approach by performing frequent interim analyses without increasing the probability of erroneous conclusions.

**Conclusion:**

This paper describes a translatable framework using simulation to support the design of prospective IPD meta-analyses.

**Supplementary Information:**

The online version contains supplementary material available at 10.1186/s12874-022-01813-4.

## Background

With its rapid spread, mutation, and unpredictable local outbreaks, COVID-19 continues to pose a global threat [1, 2]. Since early 2020, COVID-19 has led to over six hundred million cases and over six million deaths [3]. To date, there are still only a few reliable treatments, especially for the new viral variants, for which some existing medications are less or not effective at all [4]. To alleviate this emergency, researchers have initiated numerous clinical trials to find treatments. To date, over 8,000 trials have been launched worldwide for COVID-19, including over 4,000 interventional studies and randomized controlled trials (RCTs) [5]. As the pandemic surged and waned in various parts of the world, these trials were often launched in local settings after the pandemic had peaked in those regions [6]. As a consequence of a decline in new COVID-19 cases, some RCTs were terminated early [7–10], and with a lack of sufficient numbers of patients, many reported inconclusive findings. Other trials opted to pause and wait for the second and third COVID-19 surge in their regions, thus delaying conclusive findings [11]. Without a steady enrollment of trial patients, determining the efficacy and safety of treatments could require additional COVID-19 surges at the same location over several years [6].

Pooling data from ongoing and terminated RCTs holds promise for identifying effective treatments quickly [6, 12]. While conventional meta-analyses pool data from completed RCTs [13, 14], another productive approach involves synthesizing evidence from a collection of both ongoing and completed RCTs [15]. Researchers at New York University (NYU) initiated the COntinuous Monitoring of Pooled International Trials of ConvaLEscent Plasma for COVID-19 Hospitalized Patients (COMPILE) project [16]. The COMPILE study aimed to pool individual patient data (IPD) from ongoing, paused, prematurely-terminated, or completed RCTs assessing the efficacy of COVID-19 convalescent plasma (CCP) in hospitalized patients not on mechanical ventilation at the time of randomization. The goal was to engage RCTs from around the world and to monitor the continuously accumulating data for compelling evidence of CCP’s effect in order to obtain answers as soon as possible [17]. Unlike more traditional meta-analyses [18], COMPILE was designed in the presence of considerable uncertainties. The number of RCTs, number of sites within each RCT, number of RCTs within different control conditions, number of patients, the COMPILE study duration, frequency, and timing of interim looks were all unknown. These uncertainties made it difficult to use off-the-shelf statistical methods for analysis and monitoring.

Goldfeld et al. [19] proposed a statistical analysis and interim monitoring plan specifically for the COMPILE study, which included the analytic models and the rules for stopping the study. The actual data analysis of the COMPILE study was published in [17]. In addition, we used patient data from the COMPILE study to study the heterogeneous treatment effects of CCP in [20]. The current paper describes the development process of the analytic models and stopping rules presented in [19] and applied in the COMPILE study. In doing so, we provide a general, translatable framework for developing analytic models and monitoring plans for prospective IPD meta-analyses under uncertainty, which, we believe might become a common practice at least in the face of new pandemics.

Our framework is based on a Bayesian clinical trial paradigm [21, 22]. We use Bayesian hierarchical modeling [23] that explicitly accounts for patient heterogeneity and allows for “borrowing of information” across the trials [24]. This approach enables the implementation of complex statistical models with a variety of hierarchical assumptions (e.g., the number of RCTs, sites, and control conditions) and the reduction of the uncertainty of parameter estimates. Additionally, properly designed Bayesian monitoring allows continuous monitoring without the penalties for multiple interim looks and alpha-spending associated with the frequentist monitoring approach, making it an attractive and efficient strategy [24–27]. The proposed framework is not limited to the COMPILE study but is a translatable tool to guide the design of prospective IPD meta-analyses.

We organize the paper as follows. In the Methods section, we discuss methods of building a Bayesian model for quantifying a treatment’s efficacy. We describe how we extended the Bayesian model to accommodate multi-site RCTs and evaluate heterogeneous treatment effects. We outline criteria for selecting prior distribution assumptions and solutions to improve model stability. We describe the proposed goodness-of-fit methodology using posterior predictive checking. We introduce the Bayesian monitoring rules calibrated for type 1 error rate and power. In the Results section, we present our process based on extensive simulations that enabled us to finalize our analysis plan, with an eye towards understanding the impact of the prior distribution assumptions on both posterior estimations and model stability. In addition, we show the simulation results of the proposed goodness-of-fit methodology and provide details about how we set the stopping rules using type 1 error rate and power. In the Discussion and conclusion section, we conclude with a brief discussion and offer a glimpse into the future applications of our work.

## Methods

The first step in developing an analytic plan is to determine the functional form of the model, which is largely determined by the study’s primary outcome (e.g., a binary outcome suggests a logistic regression model), but also depends on the nature of the intervention as well as the hierarchical structure suggested by the study design. We started with the simplest assumptions that there would be *K* RCTs in the prospective meta-analysis and each RCT would be conducted in a single site. We also assumed that each of the *K* RCTs would have $n_k$ subjects, $k=1,\dots ,K$.

### General model for estimating across-study treatment effect

Conceptualizing this project, we started with the most general model that would allow us to estimate RCT-specific treatment effects along with RCT-specific random effects or intercepts while adjusting for patient-level covariates in case there were substantial differences across RCTs. In this model, $Y_{ki}$ denotes the outcome for the $i^{th}$ patient from the $k^{th}$ RCT. $Z_{ki}$ indicates the treatment assignment for the $i^{th}$ subject in the $k^{th}$ RCT; $Z_{ki} = 1$ if the patient is randomized to the experimental treatment arm, $Z_{ki} = 0$ if the patient is randomized to the control treatment arm. $\varvec{X}_{ki}$ denotes a vector of covariates of length *p*. The expected value of the outcome is related to the linear combination of covariates and treatment assignment via a known link function *g*(.):

1 $$\begin{aligned} g \left( E\left( Y_{ki} \right) \right)= & {} \tau _{k} + \varvec{\beta } \varvec{X}_{ki} + \theta _{k} Z_{ki}\nonumber \\ \tau _k\sim & {} \text {Normal }(\mu = 0, \sigma = \sigma _\tau )\nonumber \\ \varvec{\beta }\sim & {} \text {Normal } \left( \varvec{\mu } = \varvec{0}, \Sigma = \sigma _\beta ^2 I_{p \times p} \right) \nonumber \\ \theta _{k}\sim & {} \text {Normal }\left( \mu = \Theta , \sigma = \sigma _{\theta } \right) \nonumber \\ \Theta\sim & {} \text {Normal } \left( \mu = 0, \sigma = \sigma _\Theta \right) , \end{aligned}$$

where $\tau _{k}$ represents the RCT-specific intercept, $\varvec{\beta }$ is a vector of coefficients for the *p* covariates, and $\theta _{k}$ is the main effect of experimental treatment for the $k^{th}$ RCT. To avoid potential estimation problems related to small final and/or interim sample sizes in individual participating RCTs, the covariate effects $\boldsymbol\beta$ were not modelled as RCT-specific, unlike the treatment effect $\theta_k$ and the study-specific intercept $\tau _k$. We were agnostic at this point as to whether the prior distributions would be *Normal*, *Cauchy*, or a $t_{student}$ distribution with 3 degrees of freedom, which is a compromise between the *Normal* and *Cauchy* distributions. The variance assumptions for the prior distributions were also an open-ended target. The prior distributions for all parameters and hyperparameters were determined in later models.

A key feature of the model is that the prior distribution assumes each RCT-specific “experimental treatment effect” $\theta _{k}$ is centered around an overall effect of the experimental treatment $\Theta$. $\Theta$ represents the treatment-control effect contrast: $E(g(E(Y_{ki}\vert \boldsymbol{X}_{ki},Z_{ki}=1))-g(E(Y_{ki}\vert \boldsymbol{X}_{ki},Z_{ki}=0)))$ across all RCTs. This treatment-control effect contrast is the parameter of interest in clinical trials. In the case that the outcome is binary, for example, and *g*(.) is a *logit* link function, $\Theta$ would correspond to the experimental arm’s treatment effect on the outcome as measured by log odds ratio $(\log \mathrm {OR})$.

### General model for comparing single treatment against multiple control types

It quickly became apparent that our general approach would be inadequate, as we learned that the individual trials were likely to have varying control conditions. To accommodate this, we adjusted the model so that the experimental treatment represented the reference category. So, $A_{ki} = 0$ if the patient is randomized to the experimental arm, $A_{ki} = 1$ otherwise and we assumed there would be *C* control types in total ($C > 1$).

The updated model was specified as follows:

2 $$\begin{aligned} g \left( E\left( Y_{ki} \right) \right)= & {} \tau _{k} + \varvec{\beta } \varvec{X}_{ki} + \delta _{k_c} A_{ki}\nonumber \\ \tau _k\sim & {} \text {Normal }(\mu = 0, \sigma = \sigma _\tau )\nonumber \\ \varvec{\beta }\sim & {} \text {Normal } \left( \varvec{\mu } = \varvec{0}, \Sigma = \sigma _\beta ^2 I_{p \times p} \right) \nonumber \\ \delta _{k_c}\sim & {} \text {Normal }\left( \mu = \delta _c, \sigma = \eta \right) , \ \ c \in (1,\dots , C)\nonumber \\ \eta\sim & {} \text {Cauchy }\left( \mu = 0, \sigma = \sigma _\eta \right) , \ \ \eta \ge 0\nonumber \\ \delta _c\sim & {} \text {Normal}\left( \mu = -\Delta , \sigma = \eta _0 \right) \nonumber \\ \eta _0\sim & {} \text {Cauchy }\left( \mu = 0, \sigma = \sigma _{\eta _0} \right) , \ \ \eta _0 \ge 0\nonumber \\ -\Delta\sim & {} \text {Normal } \left( \mu = 0, \sigma = \sigma _\Delta \right) . \end{aligned}$$

The parameters $\tau _k$ and $\varvec{\beta }$ mirror the same parameters in model (1). $\delta _{k_c}$ is the main effect of *control* treatment for the $k^{th}$ RCT (i.e., RCT-specific “control effect”). Since the RCTs have the same experimental treatment but have different control types, *c* denotes the control type for the $k^{th}$ RCT, where $c \in (1,\dots ,C)$. The prior distribution assumes each RCT-specific “control effect” $\delta _{k_c}$ is centered closely around its pooled “control effect” $\delta _c$. We have introduced a hyperparameter $\eta$ that represents the study variation around the control-type mean; this hyperparameter has its own prior distribution, and we need to provide an assumed standard deviation $\sigma _\eta$. In turn, the $\delta _c$’s are assumed to center around an overall effect of control treatment $-\Delta$. We use $-\Delta$ to represent the control-treatment effect contrast: $E(g(E(Y_{ki}\vert \boldsymbol{X}_{ki},A_{ki}=1))-g(E(Y_{ki}\vert \boldsymbol{X}_{ki},A_{ki}=0)))$ across all RCTs, and $\Delta$ represents the treatment-control effect contrast: $E(g(E(Y_{ki}\vert \boldsymbol{X}_{ki},A_{ki}=0))-g(E(Y_{ki}\vert \boldsymbol{X}_{ki},A_{ki}=1)))$ across all RCTs. $\Delta$ is the parameter of interest in clinical trials. The variation around $-\Delta$ introduces a second hyperparameter $\eta _0$; we would need to provide an assumed standard deviation $\sigma _{\eta _0}$. At this point, we set prior distributions for $\eta$ and $\eta _0$ as they are of paramount importance, while $\sigma _{\tau }$, $\sigma _{\beta }$, $\sigma _{\eta }$ and $\sigma _{\eta _0}$ have point mass prior distribution as they are nuisance parameters. In evaluating evidence for efficacy, a point-mass prior of $\sigma _{\Delta }$ is often used [28, 29].

### Basic model for ordinal outcome

The COMPILE model was ultimately developed to analyze an ordinal outcome measure (COVID-19 clinical status) proposed by the World Health Organization (WHO) and widely adopted by RCTs around the world to evaluate therapies for COVID-19. This clinical status scale helps RCTs measure COVID-19 severity, with values ranging from 0 = uninfected to 10 = dead (see Additional file 1); larger values on this scale indicate more severe disease [30]. We determined that a cumulative proportional odds (*co*) model would be most appropriate for the ordinal outcome measure [31].

Our goal was to develop a *co* model that reflected the structure of the general model (2) that we were interested in. As before, we assumed there would be *K* RCTs, each of which would be using one of *C* possible control conditions. All RCTs have the same experimental treatment: CCP. The ordinal outcome for the $i^{th}$ patient from the $k^{th}$ RCT is denoted by $Y_{ki}=y$, $y=0,\dots ,10$, and the cumulative probability is $p_{kiy}=P\left( Y_{ki}\ge y\right)$. As before, $A_{ki} = 0$ if the patient is randomized to CCP arm, $A_{ki} = 1$ otherwise.

The basic version of our *co* model was specified as follows:

3 $$\begin{aligned} \text {logit} \left( P\left( Y_{ki} \ge y\right) \right)= & {} \tau _{yk} + \varvec{\beta } \varvec{X}_{ki} + \delta _{k_c} A_{ki} \nonumber \\ \tau _{yk}\sim & {} \text {Normal } \left( \mu = 0, \sigma = \sigma _\tau \right) \nonumber \\ \varvec{\beta }\sim & {} \text {Normal } \left( \varvec{\mu } = \varvec{0}, \Sigma = \sigma _\beta ^2 I_{p \times p} \right) \nonumber \\ \delta _{k_c}\sim & {} \text {Normal }\left( \mu = \delta _c, \sigma = \eta \right) , \ \ c \in (1,\dots , C) \nonumber \\ \eta\sim & {} \text {Cauchy }\left( \mu = 0, \sigma = \sigma _\eta \right) \nonumber \\ \delta _c\sim & {} \text {Normal}\left( \mu = -\Delta _{co}, \sigma = \eta _0 \right) \nonumber \\ \eta _0\sim & {} \text {Cauchy }\left( \mu = 0, \sigma = \sigma _{\eta _0} \right) \nonumber \\ -\Delta _{co}\sim & {} \text {Normal } \left( \mu = 0, \sigma = \sigma _{\Delta _{co}} \right) . \end{aligned}$$

The *co* model differs from the general model (2) in several respects. The $\tau _{yk}$’s represent the RCT-specific intercepts, for $y=1,\dots ,10$. For any specific RCT *k*, the $\tau _{yk}$’s satisfy the monotonicity requirements for the intercepts of the *co* model. Since CCP treatment is the reference, the log-odds defined from the cumulative probabilities of the CCP arm are estimated by the $\tau _{yk}$’s. $\varvec{\beta }$ is a vector of coefficients for the *p* covariates. $\delta _{k_c}$ is the $k^{th}$ RCT-specific “control effect”, measured as a $\log \mbox{OR}$. Because the RCTs have the same experimental treatment arm of CCP but have different control treatment arms, *c* denotes the control treatment type: standard of care (SOC) alone without any transfusion, $c=1$; SOC plus non-convalescent plasma, $c=2$; SOC plus saline solution, $c=3$. The prior distribution assumes each RCT-specific “control effect” $\delta _{k_c}$ is centered closely around a pooled “control effect” $\delta _c$, the corresponding type *c* control effect against CCP. The variation of each RCT effect around the groups’ mean $\delta _c$ is $\eta$, estimated from the data. In turn, the $\delta _c$’s are assumed to center around $-\Delta _{co}$, the negative of the overall study-wise effect size. $\Delta _{co}$, the key parameter of interest, represents the pooled cumulative $\log \mbox{OR}$ across all RCTs. We use $-\Delta _{co}$ so that $\Delta _{co}$ will correspond to the conventional difference of log-odds for CCP minus log-odds for control, rather than control minus CCP.

### Extended models for ordinal outcome

We explored two major extensions of the basic model (3). First, we anticipated that some RCTs would be conducted at multiple sites, and we were interested in the model that included this added level of variation. Second, we expected that heterogeneity of treatment effect might be essential, so we explored another extension that incorporated an interaction term between the treatment and a pre-specified covariate.

#### Extended model for multi-site RCTs

We assumed that there would be *K* RCTs again, but *M* total sites, where $M>K$. The outcome for the $i^{th}$ patient from the $k^{th}$ RCT and the $m^{th}$ site is denoted by $Y_{kmi}=y$, $y=0,\dots ,10$.

4 $$\begin{aligned} \text {logit} \left( P\left( Y_{kmi} \ge y\right) \right) = {\tau _{ykm}} + \varvec{\beta } \varvec{X}_{kmi} + \delta _{m_k} A_{kmi}. \end{aligned}$$

The notation largely follows model (3). The extended model (4) incorporates new parameters: $\tau _{ykm}$, and $\delta _{m_k}$. The $\tau _{ykm}$ indicates the site-specific intercept and $\delta _{m_k}$ is the $m^{th}$ site-specific “control effect”. Each $\delta _{m_k}$ is normally distributed around a RCT-specific “control effect” $\delta _{k_c}$, with a standard deviation $\eta _1$. More details of this extended model are in Additional file 2.

#### Extended model for assessing heterogeneity of treatment effect

To explore the impact of a pre-treatment covariate on the CCP effect, we developed another extension to model (3) for investigating the interaction between treatment and a categorical pre-treatment covariate, denoted by *S*. $\Delta _s$ denotes the pooled effect of CCP (measured by $\log \mathrm {OR}$) for patients with covariate $S=s$. More details of this extended model are in Additional file 3.

### Criteria for selecting prior distribution assumptions

In planning for the COMPILE analysis, it was key to establish the values of the standard deviation parameters (e.g., $\sigma _\tau$, $\sigma _\beta$, $\sigma _\eta$, $\sigma _{\eta _0}$, $\sigma _{\Delta _{co}}$ in the *co* model (3)) as well as the prior distribution families to optimize the models with respect to bias and model stability. Below is illustrated how we identified the most appropriate model parameterization, prior distribution assumptions, and coding implementation strategies. At each stage of development of the statistical analysis plan, we conducted a series of simulations to assess the models under different conditions by varying effect sizes, the numbers of RCTs within each control condition, and the number of patients.

We considered the following specific criteria for choosing prior distributions:

**Prior predictive checking**: Prior predictive checking is a standard method to determine whether an assumed prior distribution is appropriate [23]. In particular, we used prior predictive checking described in [32] to ensure that all plausible values of the outcome (e.g., WHO 11-point scale) occurred with some probability. Because the WHO clinical status scale is ordinal and not continuous, this criterion was consistently satisfied across all assumed prior distributions.

**Bias of estimated posterior distributions**: If the sample size of the simulated study is large enough, an appropriate prior distribution should produce an estimated posterior distribution consistent with the data generation process. To assess this, we generated data sets under different scenarios for the effect size, and for each scenario, we generated 2500 studies each with a total of 900 patients. While the Bayesian analysis can provide the *full* posterior distribution of the parameter of interest based on each simulated study (in this case, $\Delta _{co}$), it is challenging to compare models based on thousands of posterior distributions. Rather, we opted to use a single summary statistic, the posterior median, as the basis for comparison. For each effect size scenario, we constructed the distribution of posterior medians based on the 2500 simulated studies. An appropriate prior should result in a distribution of posterior medians that is centered around the true value used for the data generation.

**Model stability**: The Bayesian models were implemented in Stan software, which provides Bayesian inference over the model conditioned on data using Hamiltonian Monte Carlo (HMC) sampling. By default, the inference engine used is the No-U-Turn sampler (NUTS), an adaptive form of HMC sampling [33]. Divergent transitions that occur in the context of HMC sampling can lead to unreliable estimation of the posterior distributions. This results when the step size in the HMC sampling is too large to capture the highly varying posterior curvature [34]. Both model implementation and poorly conceived prior distribution assumptions can lead to undesirable levels of model divergence. The *proportion* of divergent transitions during HMC sampling is a widely used measure of stability and convergence. A model with a lower divergence rate is considered more reliable [35, 36].

Stable model estimation in Stan depends on two key tuning parameters: *adapt_delta* and *max_treedepth*. *adapt_delta* is the target average proposal acceptance rate applied during the model adaptation period; increasing this value results in a smaller step size for this gradient-based simulation of the Hamiltonian algorithm, allowing better exploration of the sample space [34]. The downsides are two-fold: (i) sampling tends to be slower because a smaller step size means that more steps are required to explore the posterior distribution thoroughly, and (ii) when the step size is too small, the sampler becomes inefficient, and the NUTS may stop before making a U-turn. But, we were able to mitigate these issues by increasing the second tuning parameter, *max_treedepth* [34].

### Goodness-of-fit using posterior predictive checking

Any consumer of a statistical model likely will ask if the applied model is a good representation of the observed data. This is particularly important when the model in question, like a *co* model, makes a strong assumption. In this case, the model makes an assumption of proportional cumulative odds. In anticipation of potential deviations from the assumptions, researchers can simulate data under a range of possible violations of the model’s assumptions and use posterior predictive checking to examine each model’s *goodness-of-fit*.

Posterior predictive checking is a powerful method to assess a model’s *goodness-of-fit* [37, 38]. The idea behind this technique is simple: if a model is a good fit, we should be able to use the model to generate replicated data ($D^{\text {rep}}$) that resemble our observed data ($D^{\text {original}}$) [39]. The lack of fit can be measured by the Bayesian *p*-value, which is the probability that the test statistic (e.g., $P(Y \le y), y=0,\dots , 9$) for $D^{\text {rep}}$ is equal to or greater than the test statistic for $D^{\text {original}}$ [23]. A Bayesian *p*-value very close to zero or one is a cause for concern that the model is not fitting the data well, while a Bayesian *p*-value close to 0.5 means the model captures the data well [23, 33]. The procedure for checking whether the *co* model fits the observed data well and for calculating the Bayesian *p*-value can be found in Additional file 4.

### Interim monitoring for efficacy

COMPILE pre-specified co-primary endpoints, both based on the WHO 11-point scale: the WHO 11-point ordinal scale, and a binary indicator of WHO $\ge$ 7. These two outcomes accommodate two essential functions: efficiency and interpretability. This section introduces the stopping rule based on the two outcomes.

#### Basic model for binary outcome

The second primary outcome selected with the goal of ease of clinical interpretation, is derived from the WHO 11-point clinical status scale and indicates that the patient is on mechanical ventilation or worse, i.e., WHO $\ge 7.$ We determined that a logistic (*l*) model would be most appropriate for the binary outcome. The *l* model was included for ease of communication and acceptability by the clinical community.

In model (5), *W* is an indicator variable for a WHO score $\ge 7$, $W=1$ if the patient has a WHO score $\ge$ 7, and $W=0$ otherwise.

5 $$\begin{aligned} \text {logit} \left( P\left( W_{ki} = 1\right) \right) = \tau _{k} + \varvec{\beta } \varvec{X}_{ki} + \delta _{k_c}A_{ki} \end{aligned}$$

The parameters of model (5) mirror the parameters in model (3). The primary parameter of interest is $\Delta _l$, the overall effect of CCP compared to any control.

#### Interim monitoring

In discussions with experts in the fields of RCTs, Bayesian analysis and monitoring of RCTs, conditions for stopping the COMPILE study were identified. The stopping rules were based on the following posterior probabilities for the $\mathrm {ORs}$ ($\mathrm {OR}_{co} = e^{\Delta _{co}}$ and $\mathrm {OR}_{l} = e^{\Delta _{l}}$):

6 $$\begin{aligned} P\left( \mathrm {OR}_{co}< 1 \right) \ge 0.95& \qquad \& &P\left( \mathrm {OR}_{co}< 0.8 \right) \ge 0.50 \nonumber \\&\qquad \text {and}&\nonumber \\ P\left( \mathrm {OR}_{l}< 1 \right) \ge 0.95&\qquad \& &P\left( \mathrm {OR}_{l} < 0.8 \right) \ge 0.50 \end{aligned}$$

When $\mathrm {OR}_{co} < 1$ and $\mathrm {OR}_{l} < 1$, CCP is at least minimally more effective than control; we required a high level of certainty that this be the case. When $\mathrm {OR}_{co} < 0.8$ and $\mathrm {OR}_{l} < 0.8$, it is considered that the beneficial effect of CCP is more than trivial; we required a moderate level of certainty that this be the case.

The stopping rules pertained to the monitoring of COMPILE study and the execution of the COMPILE meta-analysis itself, and had no direct bearing on the conduct of the individual studies. During the pandemic, the rapid dissemination of high-quality information was viewed as paramount, so once the criteria were met for stopping the prospective meta-analysis study, COMPILE data collection ceased, the final analyses were conducted, and results were published; the individual studies could have chosen to continue to enroll patients or suspend enrollment and continue only to follow up patients already enrolled.

The goal of the COMPILE was to provide answers regarding the efficacy of CCP treatment as soon as possible. Since neither the number of interim looks nor the number of RCTs and number of patients at each interim look could be predicted when we were planning the study, extensive simulations were required to calibrate the Bayesian criteria against the frequentist standards for type 1 error rates and statistical power.

## Results

In order to finalize our analysis plan, we used extensive simulations to evaluate and choose prior distribution assumptions for both the basic and the extended models. We also used simulation to validate our proposed method of assessing goodness-of-fit using posterior predictive checking as well as assess the operating characteristics of the proposed Bayesian stopping rules for efficacy.

### Evaluating and choosing prior distribution assumptions for the basic model

We started with an initial set of prior distribution assumptions (labeled as *Version 1*) for the parameters in the basic model (3). Using simulations, we evaluated the suitability of this version of assumptions with respect to the criteria described in the section titled Criteria for selecting prior distribution assumptions. Based on the findings from these simulations, we updated and reevaluated a new set of prior distribution assumptions. We iterated through this process a number of times until we were satisfied that the criteria were reasonably met. The sequential versions are shown in Table 1.

**Table 1:** Prior distributions for different versions of cumulative proportional odds model

Versions	1	2	3	final
$\alpha$	0	0	0	Normal ($\mu = 0,\ \sigma = 0.1$)
$\tau _{yk}$	Normal ($\mu = 0,\ \sigma = 100$)	Normal ($\mu = 0,\ \sigma = 100$)	Normal ($\mu = 0,\ \sigma = 100$)	$t_{\text {student}} (\mathrm {df} = 3,\ \mu = 0,\ \sigma = 8)$
$\boldsymbol{\beta}$	Normal ($\boldsymbol\mu=\mathbf0,\mathrm\Sigma=100^2I_{p\times p}$)	Normal ($\boldsymbol\mu=\mathbf0,\mathrm\Sigma=100^2I_{p\times p}$)	Normal ($\boldsymbol\mu=\mathbf0,\mathrm\Sigma=100^2I_{p\times p}$)	Normal ($\boldsymbol\mu=\mathbf0,\mathrm\Sigma=2.5^2I_{p\times p}$)
$\delta _{k_{c}}$	Normal ($\mu = \delta _{c},\ \sigma = \eta$)	Normal ($\mu = \delta _{c},\ \sigma = \eta$)	Normal ($\mu = \delta _{c},\ \sigma = \eta$)	Normal ($\mu = \delta _{c},\ \sigma = \eta$)
$\eta$	Cauchy ($\mu = 0,\ \sigma = 100$)	$t_{\text {student}} (\mu = 0,\ \sigma = 100)$	$t_{\text {student}} (\mu = 0,\ \sigma = 100)$	$t_{\text {student}} (\mathrm {df} = 3, \mu = 0,\ \sigma = 0.25)$
$\delta _{c}$	Normal ($\mu=-\triangle_{co},\;\sigma=\eta_0$)	Normal ($\mu=-\triangle_{co},\;\sigma=\eta_0$)	Normal ($\mu=-\triangle_{co},\;\sigma=\eta_0$)	Normal ($\mu=-\triangle_{co},\;\sigma=\eta_0$)
$\eta_0$	Cauchy ($\mu = 0,\ \sigma = 100$)	$t_{\text {student}} (\mu = 0,\ \sigma = 100)$	$t_{\text {student}} (\mu = 0,\ \sigma = 100)$	0.1
$-\triangle _{co}$	Normal ($\mu = 0,\ \sigma = 100$)	Normal ($\mu = 0,\ \sigma = 100$)	Normal ($\mu = 0,\ \sigma = 0.354$)	Normal ($\mu = 0,\ \sigma = 0.354$)

#### Simulation setup - basic model

The evaluation was conducted using the R package *simstudy* [40] to generate simulated data sets with the following parameters:

- We assumed different effect sizes for the three different control types. The overall effect $\Delta _{co}$ was set at the simple negative average of the three $\delta _c$’s :
  - $$\delta _1 = 0.3$$
  - $$\delta _2 = 0.4$$
  - $$\delta _3 = 0.5$$
  - $$\Delta _{co} = -0.4$$
- The between study and within control type variation was set at $\sigma = 0.1$
- We assumed three RCTs within each control type, with the size of the RCTs being
  - 1 large RCT with $n=150$
  - 2 small RCTs, each with $n=75$
- We started with a total sample size 900 as this was our initial aspiration for the COMPILE study

For each simulated individual, we generated a set of covariates: age (a categorical variable with 1 indicating < 50 years old, 2 indicating [50,65), and 3 indicating $\ge$ 65 years old), gender (a binary variable defined as female and male), WHO score at baseline (an ordinal variable with possible values of 4, 5, and 6) and duration of symptoms before randomization (a categorical variable with 1 for 0-3 days, 2 for 4-6 days, 3 for 7-10 days, 4 for 11-14 days, and 5 for 14+ days). We generated the distributions of these baseline covariates based on data available from the first RCT that joined the COMPILE consortium. In the simulation, we assumed the following distribution of baseline covariates: **age** < 50 years old : [50, 65) : $\ge$ 65 years old = 1 : 1 : 2; **sex** = female : male: = 1 : 1; **baseline WHO score** = 4 : 5 : 6 = 1 : 1 : 1; **duration of symptoms before randomization** = 0-3 days : 4-6 days : 7-10 days : 11-14 days : 14+ days = 1 : 1 : 1 : 1 : 1. The ordinal WHO outcome was generated as a function of the RCT-specific intercept, the individual-level covariates, and a random treatment assignment. We selected the coefficients from both the available COMPILE RCT data and from the literature describing outcomes of COVID-19 — male, older, and patients with higher severity of symptoms at baseline and patients with longer duration of symptoms were at higher risk for worse outcome. We present the coefficients of the covariates as “true values” in Additional file 5.

We simulated 2500 trials (each trial included 9 RCTs) for model fitting. For each simulated trial, we used 2000 HMC iterations for warm-up and retained 10000 iterations for inference (all simulations in this paper used the same number of HMC iterations. See code for simulations in Additional file 6.).

**Fig. 1:**
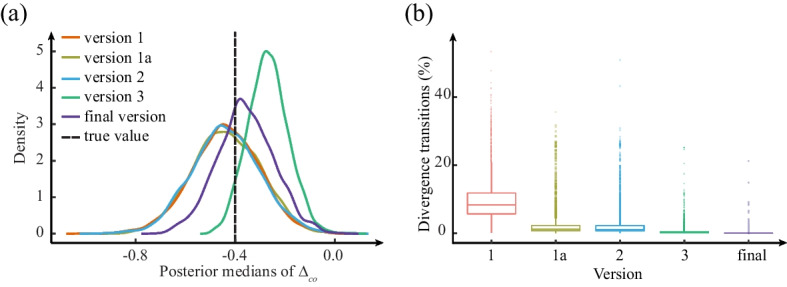
The performance of the five versions of the basic *co* model: **a** the distribution of posterior medians of the pooled CCP treatment effect $\Delta _{co}$ and the true value used to generate the data (true value); **b** boxplots show the median, lower quartile, upper quartile, minimum, and maximum of the number of divergent transitions ($\%$). The proportion of divergent transitions was calculated by (the number of divergent transitions/10,000)$\times 100\%$ in each simulated trial using the five versions of model

Figure 1 shows the bias of the posterior estimations as well as the divergences resulting from each set of modelling assumptions based on our simulating assumptions. The bias is based on a comparison of the center of the distribution of posterior medians with the “true value” of $\Delta _{co}= -0.4$; the greater the difference, the greater the bias.

#### Models Version 1 and 1(a)

Version 1 was based largely on non-informative prior distribution assumptions [41]. The proportion of divergent transitions from the simulations was unacceptably high, indicating that the posterior estimation from this model was unlikely to be reliable (see Fig. 1(b)).

Version 1(a) was unchanged from Version 1, except that we used non-centered parameterization to implement the model in Stan. Non-centered parameterization is an additional tool to improve model fitting, and the *Normal* distribution is the best candidate for reparameterization [33]. Based on this, we applied the non-centered parameterization for all *Normal* prior distributions in the model. While the proportion of divergent transitions decreased relative to Version 1, the reduction was not sufficient to ensure model stability (see Fig. 1(b)).

#### Model Version 2

In the second version of the model, we replaced the *Cauchy* distributions with $t_{student}$ distributions, which are more suitable as prior distributions of the scale parameters [42]. The $t_{student}$ distribution with 3 degrees of freedom ($\sigma = 100$) has tails that present a compromise between the *Normal* distribution ($\sigma = 100$) and the *Cauchy* distribution ($\sigma = 100$). This change resulted in fewer divergent transitions compared to Version 1(a), but again the reduction was insufficient (see Fig. 1(b)).

#### Model Version 3

In the third iteration, we imposed a skeptical prior on $-\Delta _{co}$ to reflect a very conservative belief in the efficacy of the treatment. A skeptical prior distribution assumes that the probability of large benefit or harm from the experimental treatment is low and that the probability of equivalence between the treatments is high. The standard deviation of the prior $(\sigma _{\Delta _{co}})$ was set at 0.354, which corresponds to a prior for an $\mathrm {OR}$ with 95% density between 0.5 and 2. With this prior distribution, the effect of CCP is assumed to be close to zero, and only strong evidence from the data can alter this prior belief. When assessing evidence of efficacy, such a skeptical prior for the treatment effect is considered appropriate [24, 28, 29]. However, the distribution of posterior medians from posterior estimates of the overall effect did not adequately reflect the “true” underlying data generation process (see Fig. 1(a)).

#### Final Version

We settled on this final version of the *co* model after the sequence of simulation experiments:

7 $$\begin{aligned} \text {logit} \left( P\left( Y_{ki} \ge y\right) \right)= & {} \alpha + \tau _{yk} + \varvec{\beta } \varvec{X}_{ki} + \delta _{k_c} A_{ki}\nonumber \\ \alpha\sim & {} \text {Normal } (\mu = 0, \sigma = 0.1)\nonumber \\ \tau _{yk}\sim & {} t_{\text {student}} \left( \text {df}= 3, \mu = 0, \sigma = 8 \right) \nonumber \\ \varvec{\beta }\sim & {} \text {Normal } \left( \varvec{\mu } = \varvec{0}, \Sigma = 2.5^2 I_{p \times p} \right) \nonumber \\ \delta _{k_c}\sim & {} \text {Normal }\left( \mu = \delta _c, \sigma = \eta \right) \ \ c \in (1, 2, 3)\nonumber \\ \eta\sim & {} t_{\text {student}}\left( \text {df} = 3, \mu = 0, \sigma = 0.25 \right) \nonumber \\ \delta _c\sim & {} \text {Normal }\left( \mu = -\Delta _{co}, \sigma = 0.1 \right) \nonumber \\ -\Delta _{co}\sim & {} \text {Normal } \left( \mu = 0, \sigma = 0.354 \right) \end{aligned}$$

The following updates were made to Version 3.

**Global intercept** $\alpha$: $\alpha$ is a nuisance parameter that we expected to be very close to or at 0. Since we modelled each RCT-specific intercept directly, $\alpha$ was effectively fixed to 0 in Version 3. However, model fitting improved when $\alpha$ was freely estimated. In the final version of the model, we used a highly informative prior where most of the probability mass was set close to zero.

**RCT-specific intercepts** $\tau _{yk}$: We initially assumed that the prior distribution for each $\tau _{yk}$ was $Normal(\mu =0,\sigma =100)$. The domain experts participating in the study suggested that we use a weakly-informative prior distribution with scale parameter = 8 to act as somewhat of a constraint. However, we used a $t_{student}$ distribution with 3 degrees of freedom ($\sigma = 8$). This $t_{student}$ distribution has heavier tails than the *Normal* distribution ($\sigma = 8$), so we ensured that the HMC simulation would have enough flexibility to explore the sample space.

**Covariate coefficients** $\varvec{\beta }$: We had little prior information for $\varvec{\beta }$ and expected the observed data to determine the shape of the posterior distribution, so we assumed a diffuse prior distribution.

**Standard deviation of RCT-specific “control effect”** $\eta$: The prior distribution of $\delta _{k_c}$ is normally distributed around its own “control effect”: $\delta _{c}$ , with a standard deviation $\eta$. In the final version, $\eta$ had an informative prior distribution $t_{student}(df=3,\mu =0,\sigma =0.25)$. This $t_{student}$ distribution has heavier tails than the *Normal* distribution with equivalent scale parameters($\sigma =0.25$).

**Standard deviation of control-type effect** $\eta _0$: We consulted the domain experts involved in the study, who suggested that the three types of control conditions should not differ greatly; in particular, they believed $95\%$ of the possible values ($\log \mathrm {OR}$) should be within 0.2 of the mean, implying a standard deviation of 0.1. Thus, the solution was to use a more informative prior with narrow tails.

The simulation results shown in Fig. 1 indicate that the position with the highest probability is very close to the “true” value for data generation. Because of the postulated skeptical prior, the posterior estimation was pulled very slightly (rightwards) towards 0, despite the relatively large sample size. The number of divergent transitions was close to or at zero for nearly all simulated trials, indicating that model fitting converged almost every time.

We also assessed the models under different scenarios for the effect sizes. The results were consistent at different effect sizes ($\delta _1,\delta _2,\delta _3$). See Additional file 7 for the bias in posterior estimates and divergent transitions resulting from each version of the *co* model under a different scenario for effect sizes ($\delta _1,\delta _2,\delta _3$) = (0.05, 0.1, 0.15).

With the final model in hand, we were able to look at extended models, explore goodness-of-fit methods, evaluate operating characteristics of stopping rules, and examine the influence of the sample size we had assumed (see Additional file 8 for model fitting results of the *co* model using various sample sizes).

### Evaluating and choosing priors for the extended model

After finalizing the basic *co* model (3) as in model (7), we turned our attention to the extended models.

#### Extended model for multi-site RCTs

In the simulation setup, we assumed that there would be *K* RCTs again, but *M* total sites, where $M>K$. The outcome for the $i^{th}$ patient from the $k^{th}$ RCT and the $m^{th}$ site is denoted by $Y_{kmi}=y$, $y=0,\dots ,10$.

- 3 control types with effect sizes: $\delta _1 = 0.3$, $\delta _2$ = 0.4, $\delta _3$ = 0.5
- Between study (within control type) variation $\sigma = 0.1$
- 3 RCTs within each control type
  - 1 large RCT with  $n=150$: 1 large site with  $n=110$ and 2 small sites with  $n=20$
  - 2 small RCTs, each with  $n=75$: 1 large site with  $n=55$ and 2 small sites with  $n=10$
- Between sites (within RCT) variation $\sigma = 0.1$

**Fig. 2:**
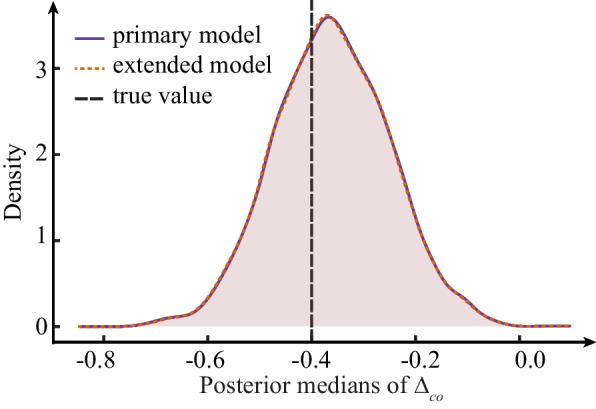
The distribution of posterior medians of pooled CCP treatment effect $\Delta _{co}$ using the final version of basic *co* model and the extended *co* model for multi-site RCTs. The black dashed line represents the true value of parameter used to generate the data

For each simulated individual, we also generated a set of covariates: age, gender, WHO score at baseline, and duration of symptoms before randomization. The randomization to CCP and control is within sites. The prior distributions for the extended model for multi-site RCTs are in Additional file 9. We conducted 3000 simulations to compare the performance of model (7) and the model for multi-site RCTs. and found that both performed well in recovering the “true value” (see Fig. 2; the estimations of all parameters can be found in Additional file 10). The posterior distributions from both models were virtually identical. Given the similarities of the model estimates, we opted for the simpler model (7) that has fewer hierarchical assumptions and less complexity.

#### Extended model for assessing heterogeneity of treatment effect

Next, we focused on selecting the prior distributions for the extended model for studying the heterogeneity of the treatment effect (model (A2) in Additional file 3), which includes a term for the interaction between treatment indicators and a categorical pre-treatment variable *S*.

In the simulation setup, the data were simulated as described in the Simulation setup - basic model section with adjustment for the same set of covariates and a categorical covariate *S* with three levels (30% patients with *S* = 1, 30% patients with *S* = 2, and 40% patients with *S* = 3), as well as the interaction between covariate *S* and treatment. The covariate *S* was not associated with the other covariates. We conducted a series of simulations to assess the models under different conditions by varying treatment’s effect sizes for the overall and for the subgroups. The prior distributions of the model with the interaction between treatment and a pre-specified covariate *S* are specified in model (A4) in Additional file 11.

**Fig. 3:**
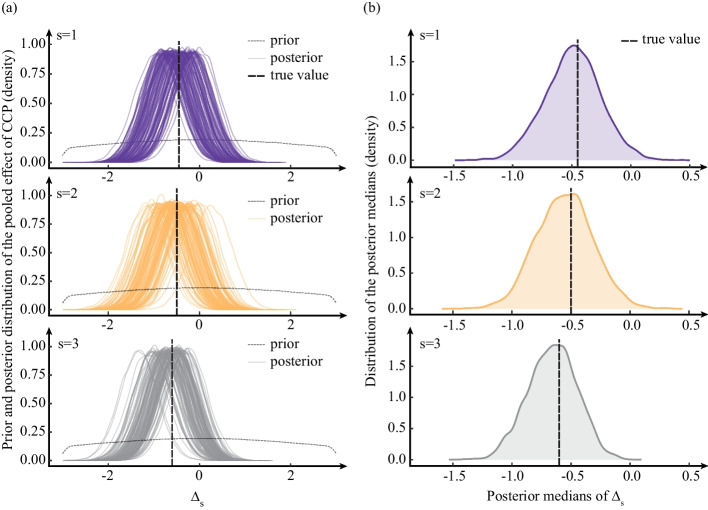
The performance of the extended model for assessing heterogeneity of treatment effect: **a** prior and 100 posterior distributions of the pooled CCP effect in each level of a pre-specified covariate ($\Delta _s$, *s* = 1,2, or 3), **b** Distribution of posterior medians of $\Delta _s$ (Based on 4500 simulated trials). The black dashed lines represent the true values of parameters used to generate the data: $\Delta _{s=1}$ = -0.45, $\Delta _{s=2}$ = -0.5, $\Delta _{s=3}$ = -0.6

Figure 3 shows the performance of the extended model for assessing the heterogeneity of treatment effect. Figure 3(a) shows the prior distribution and 100 posterior distributions of $\Delta _s$. Figure 3(b) shows the distributions of the posterior medians of $\Delta _s$ from 4500 simulated trials. The Bayesian model estimations had a very high probability of recovering “true values”.

### Simulation results for goodness-of-fit

**Fig. 4:**
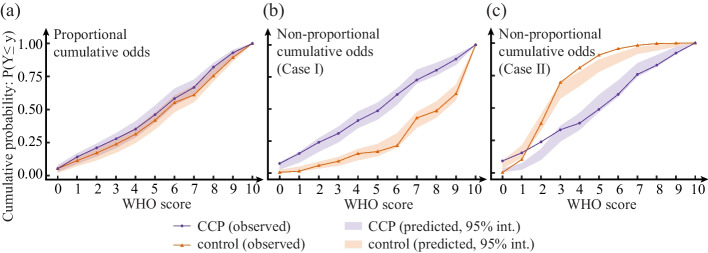
The observed cumulative probabilities for the CCP arm and the control treatment arm (CCP arm in observed data: solid purple line with marker $\bigcirc$; control arm in observed data: solid orange line with marker $\bigtriangleup$) as well as the 95% credible interval (the colored bands) for the predicted cumulative probabilities using the posterior predictive checking: **a** observed data was generated under proportional cumulative odds assumption, **b** observed data was generated when the proportional cumulative odds assumption was violated only slightly (Case I), **c** observed data was generated when the proportional cumulative odds assumption was violated more extremely (Case II)

In the case of the *co* model, there is an assumption of proportional cumulative odds. We investigated to what degree the proportional odds assumption has to be violated to render the model inaccurate. We considered two data-generating mechanisms: the observed data generated under (i) a proportional cumulative odds assumption or (ii) a non-proportional cumulative odds assumption. In addition to the case satisfying the proportionality assumptions, in Fig. 4 and Table 2 we report two cases where the proportionality assumptions were violated: one with small and one with large deviation from proportionality of the odds.

In Fig. 4, the bands represent the 95% credible interval for test statistics based on 10000 replicated datasets $D^{rep}$. The solid lines with markers represent the cumulative probabilities in $D^{\text {original}}$. Table 2 shows posterior predictive checking of model (7) using ten test statistics. We confirmed that the model fits the data well if the data generation process satisfied the proportional odds assumption. When the proportional cumulative odds assumption was violated only slightly (Case I), only one Bayesian *p*-value [23, 33] was close to one (i.e., 0.95), which would still give us confidence that our model was a good fit. However, when the proportional cumulative odds assumption was violated more extremely (Case II) in the data generation process, most Bayesian *p*-values were extreme (i.e., close to zero or one), indicating our model might be a poor fit.

**Table 2:** Summary of posterior predictive checking based on the ten test statistics

Assumption						
Treatment	CCP			Control		
Test quantity: % subjects	$T(D^{original})$	95% int. for $T(D^{rep})$	Bayesian *P* value	$T(D^{original})$	95% int. for $T(D^{rep})$	Bayesian *P* value
	**a** Proportional cumulative odds					
WHO $\le$ 0	5.32	[2.91, 7.99]	0.48	5.35	[2.18, 6.80]	0.14
WHO $\le$ 1	14.19	[8.96, 16.95]	0.24	11.36	[7.28, 14.56]	0.34
WHO $\le$ 2	21.06	[14.53, 24.21]	0.23	17.15	[11.65, 20.63]	0.32
WHO $\le$ 3	27.94	[21.07, 32.20]	0.30	23.83	[17.48, 27.91]	0.29
WHO $\le$ 4	35.25	[29.06, 41.16]	0.45	31.40	[24.76, 36.17]	0.34
WHO $\le$ 5	46.12	[40.19, 53.03]	0.53	41.87	[34.95, 47.57]	0.42
WHO $\le$ 6	58.31	[53.27, 65.86]	0.67	55.23	[48.06, 60.68]	0.40
WHO $\le$ 7	66.74	[60.77, 72.64]	0.52	61.02	[55.58, 67.96]	0.61
WHO $\le$ 8	82.04	[75.30, 85.23]	0.28	75.50	[71.36, 82.04]	0.68
WHO $\le$ 9	92.90	[88.86, 95.16]	0.35	89.31	[86.41, 93.69]	0.74
	**b** Non-proportional cumulative odds (Case I)					
WHO $\le$ 0	7.57	[2.91, 8.50]	0.07	0.67	[0.24, 2.66]	0.88
WHO $\le$ 1	15.37	[8.25, 16.50]	0.07	1.55	[1.45, 5.08]	0.95
WHO $\le$ 2	24.05	[16.75, 27.43]	0.21	5.99	[3.63, 8.96]	0.54
WHO $\le$ 3	30.96	[24.03, 36.17]	0.37	9.31	[5.81, 12.59]	0.42
WHO $\le$ 4	40.98	[35.19, 48.06]	0.55	15.30	[10.17, 18.89]	0.30
WHO $\le$ 5	48.55	[41.75, 54.85]	0.44	16.85	[13.32, 22.76]	0.65
WHO $\le$ 6	61.25	[53.40, 66.02]	0.32	21.51	[20.10, 31.23]	0.93
WHO $\le$ 7	72.61	[69.90, 80.58]	0.84	43.02	[35.35, 47.70]	0.32
WHO $\le$ 8	80.18	[76.21, 85.92]	0.65	48.56	[43.34, 55.93]	0.63
WHO $\le$ 9	88.86	[84.47, 92.23]	0.42	61.86	[56.90, 69.01]	0.64
	**c** Non-proportional cumulative odds (Case II)					
WHO $\le$ 0	9.11	[0.49, 3.16]	0.00	0.22	[3.15, 9.20]	1.00
WHO $\le$ 1	15.33	[2.43, 7.28]	0.00	10.22	[11.62, 21.07]	1.00
WHO $\le$ 2	23.78	[9.95, 18.45]	0.00	38.00	[32.69, 45.04]	0.61
WHO $\le$ 3	33.11	[24.03, 36.17]	0.16	69.56	[57.14, 69.25]	0.02
WHO $\le$ 4	38.22	[33.01, 46.12]	0.65	80.89	[66.83, 77.97]	0.00
WHO $\le$ 5	48.89	[46.84, 59.95]	0.90	90.67	[77.72, 87.17]	0.00
WHO $\le$ 6	60.44	[58.50, 71.12]	0.92	95.56	[84.75, 92.25]	0.00
WHO $\le$ 7	75.78	[74.03, 84.47]	0.92	98.22	[91.53, 96.85]	0.00
WHO $\le$ 8	82.89	[81.80, 90.53]	0.94	99.56	[94.19, 98.31]	0.00
WHO $\le$ 9	92.22	[91.26, 97.09]	0.92	99.78	[97.34, 99.76]	0.01

Note: Case I: the proportional cumulative odds assumption was violated only slightly. Case II: the proportional cumulative odds assumption was violated more extremely

### Bayesian stopping rules for efficacy

We investigated the probability of stopping early under the proposed Bayesian approach (6) using a range of effect sizes and sample sizes (Scenario (1) was simulated as in Simulation setup - basic model section: *n* = 900, Scenario (2) doubled the sample size and Scenario (3) tripled the sample size).

Parameters:

- The Bayesian paradigm includes nine data looks at 20%, 33%, 40%, 50%, 60%, 67%, 80%, 90% and 100% of the data. Three sets of control-specific treatment effects as measured by $\log \mathrm {OR}$ $(\delta _1, \delta _2, \delta _3)$ are considered:
  - (0,0,0), pooled control effect is 0
  - (0.1, 0.2, 0.3), pooled control effect is 0.2
  - (0.4, 0.5, 0.6), pooled control effect is 0.5
- No covariate adjustment in both data generation and analysis

**Fig. 5:**
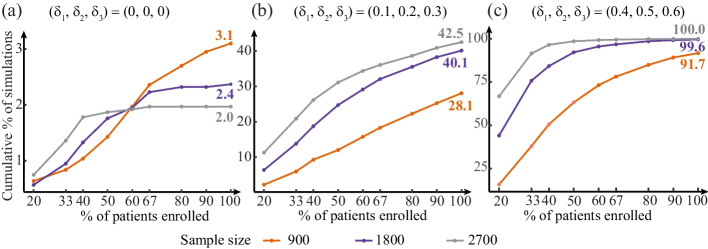
At different sample sizes, the proportion of times (out of 2000) in which the stopping rules were reached under the Bayesian monitoring approach. The colored numbers in **a** can be interpreted as the type 1 error rates at different sample sizes. The colored numbers in **b** and **c** can be interpreted as the statistical power

When the simulated effect is $(\delta _1, \delta _2, \delta _3)=(0,0,0)$, the sum of the probabilities of meeting the stopping trigger at all interim looks under the Bayesian monitoring approach can be interpreted as the type 1 error rate (see Fig. 5(a)). When data are simulated under the assumption of efficacy, the sum of the probabilities of meeting the stopping trigger (over all interim looks) can be interpreted as statistical power (see Fig. 5(b) and (c)).

The prior distributions in the model for the binary co-primary outcome WHO$\ge 7$ (model (5)) were selected through a process similar to the process for selecting the prior distributions for model (3) for the ordinal outcome. The prior distributions for the binary co-primary outcome are shown in Additional file 12:

Figure 5 shows the results for all three sets of effect sizes. The type 1 error rates were considerably below the 5% threshold. As expected, the type 1 error rate declined as the sample size increased. In the cases where CCP was assumed to be effective, larger effect sizes and larger sample sizes both increased power. We can see that the proposed Bayesian stopping rule would achieve acceptable type 1 error rates and power.

Continuous monitoring is critical in a pandemic to detect early signals of efficacy and make timely decisions. One concern with the increasing number of interim looks would be inflated type 1 error rate. In our simulation, we expected nine data looks so the stopping rules resulted in acceptable type 1 error rates and power. If researchers expect more interim looks, there are three ways to control for the inflated type 1 error rate: (i) adopt a more skeptical prior distribution for the treatment effect ($\mathrm {OR}_{co}$ and $\mathrm {OR}_l$); (ii) increase the sample size; or (iii) set a more restrictive threshold for the stopping criteria. For example, the current threshold for the second criterion of clinically meaningful effect (i.e., $P(\mbox{OR} < 0.8) \ge 0.5$) in the stopping rules is 0.5. We could increase 0.5 to 0.6 to reduce the type I error rate while keeping the prior distributions and sample size constant.

## Discussion and conclusions

The presented work describes a translatable framework for developing a rigorous plan for monitoring and analysis of a study that prospectively pools IPD from ongoing, paused, prematurely-terminate, or completed RCTs with the goal of reaching a conclusion regarding the efficacy of a treatment as quickly as possible. Such studies were in particularly high demand during the initial stages of the COVID-19 pandemic, and it is expected that they would be needed not only in future pandemics but also for contributing to more efficient non-pandemic medical research. While the idea of such prospective pooling of data from RCTs at different stages of execution is simple and appealing, the development of the analytic plan for monitoring and analysis is not trivial.

In this paper, we report on the extensive simulation investigations that were needed for selecting models and parameters to estimate and for choosing the prior distributions for these parameters. We also show how we can study the operating characteristics of guidelines for continuous monitoring in the absence of information about the total sample size, the rate of patient recruitment, and the number of interim looks at the time of study planning.

Our work should be interpreted in the context of three potential limitations. First, our extended model for assessing heterogeneity of the treatment effect (model (A4)) was designed for the interaction term between a categorical covariate and treatment. It would be useful to extend the model to incorporate the interaction term between a continuous covariate and treatment. While this is important methodologically, it may be less so clinically, since patient characteristics that are best measured using a continuous scale are routinely considered in categorical terms; viewed this way, providing interaction models only for categorical characteristics may not be such a serious limitation. Second, the 95% credible interval for the CCP treatment effect from model for assessing heterogeneity of treatment effect (model (A4)) tends to be wider than model (7) in subgroup analysis because of the diffuse prior in model (A4). Developing more efficient approaches for estimating interactions would be a valuable contribution. Third, our method focuses on sampling from the posterior distribution of the effect size $\Delta$ rather than testing the equality of experimental and control treatments, an approach that some believe is more appropriate for this setting [43]. The testing formulation, however, can require high computational overhead compared to the estimation approach we used. Regardless of whether one considers testing or estimation to be more appropriate at the stage when the prospective IPD study is completed and the final data are available, at the stage of developing the analytic plan, there may be less flexibility. Unless advances in computing make the testing approach more practical, when extensive simulations are necessary and must include a range of relatively large sample sizes (here 900, 1800 and 2700), we recommend the estimation approach described in this paper.

To the best of our knowledge, an initiative like COMPILE has not been undertaken previously. In conducting this study, we believe we have developed a translatable framework that can be used to inform such endeavors in the future. This framework can leverage information quickly for other types of therapies under simultaneous investigations around the world. Not only can this framework be a valuable tool for assessing new treatment options for COVID-19, but it can also be useful for the treatment of other diseases.

## Supplementary Information

**Additional file 1.** The WHO 11-point COVID-19 clinical status scale [30].

**Additional file 2.** Extended model for multi-site RCTs.

**Additional file 3.** Extended model for assessing heterogeneity of treatment effect.

**Additional file 4.** Procedure for posterior predictive checks [23].

**Additional file 5.** The true value of parameters in the data generation process.

**Additional file 6.** **a** R code for simulation [33, 44, 45]. **b** Stan code for the final version of the *co* model.

**Additional file 7.** Assessing models using a different set of effect sizes.

**Additional file 8.** The effect of sample sizes on the model’s performance.

**Additional file 9.** Final model for multi-site RCTs.

**Additional file 10.** Posterior estimations of parameters in the extended model for multi-site RCTs.

**Additional file 11.** Final model for assessing heterogeneity of treatment effect.

**Additional file 12.** Final model for the binary co-primary outcome.

## Data Availability

The datasets used and analysed during the current study are available from the corresponding author on reasonable request.

## Abbreviations

COVID-19: Coronavirus disease 2019
COMPILE: COntinuous Monitoring of Pooled International Trials of ConvaLEscent Plasma for COVID-19 Hospitalized Patients
IPD: individual patient data
RCT(s): randomized controlled trial(s)
SOC: standard of care
CCP: COVID-19 convalescent plasma
WHO: World Health Organization
HMC: Hamiltonian Monte Carlo
NUTS: No-U-Turn sampler
*co*: cumulative proportional odds
*l*: logistic
OR(s): odds ratio(s)
NYU: New York University
