# Supplementary material for: Developing a Bayesian hierarchical model for a prospective individual patient data meta-analysis with continuous monitoring
Source: BMC Med Res Methodol. 2023 Jan 25;23:25. doi: 10.1186/s12874-022-01813-4 (PMC9875783; doi:10.1186/s12874-022-01813-4)
Supplement: Supplementary file 1 — Additional file 1. The WHO 11-point COVID-19 clinical status scale [30]. [file 12874_2022_1813_MOESM1_ESM.pdf]

Supplementary Materials

Additional file 1 — The WHO 11-point COVID-19 clinical status scale.

0:	Uninfected, no viral RNA detected
1:	Asymptomatic, viral RNA detected
2:	Symptomatic, independent
3:	Symptomatic, assistance needed
4:	Hospitalized, no oxygen therapy
5:	Hospitalized, oxygen by mask or nasal prongs
6:	Hospitalized, oxygen by non-invasive ventilation or high flow
7:	Intubation & mechanical ventilation, $pO_2/FiO_2 \geq 150$ (or $SpO_2/FiO_2 \geq 200$)[a]
8:	Mechanical ventilation, $pO_2/FiO_2 < 150$ (or $SpO_2/FiO_2 < 200$) or vasopressors
9:	Mechanical ventilation, $pO_2/FiO_2 < 150$ and vasopressors, dialysis, or ECMO[b]
10:	Dead

[a] pO_2: partial pressure of oxygen, FiO_2: fraction of inspired oxygen, SpO_2: oxygen saturation.

[b] ECMO: extracorporeal membrane oxygenation.

Table A1 The WHO 11-point COVID-19 scale definition[30]
