# Supplementary material for: Developing a Bayesian hierarchical model for a prospective individual patient data meta-analysis with continuous monitoring
Source: BMC Med Res Methodol. 2023 Jan 25;23:25. doi: 10.1186/s12874-022-01813-4 (PMC9875783; doi:10.1186/s12874-022-01813-4)
Supplement: Supplementary file 2 — Additional file 2. Extended model for multi-site RCTs. [file 12874_2022_1813_MOESM2_ESM.pdf]

Additional file 2 — Extended model for multi-site RCTs

We assume that there are again K RCTs but M total sites, where $M > K$. The outcome for the ith patient from the kth RCT and the mth site is denoted by $Y_{kmi} = y$, $y = 0, \ldots, 10$.

$$\begin{aligned}
\text{logit}\left(P\left(Y_{kmi} \geq y\right)\right) &= \tau_{ykm} + \boldsymbol{\beta}\boldsymbol{X}_{kmi} + \delta_{m_k} A_{kmi} \\
\tau_{ykm} &\sim \text{Normal}\ \left(\mu = 0, \sigma = \sigma_\tau\right) \\
\boldsymbol{\beta} &\sim \text{Normal}\ \left(\boldsymbol{\mu} = \mathbf{0}, \Sigma = \sigma_\beta^2 I_{p\times p}\right) \\
\delta_{m_k} &\sim \text{Normal}\ \left(\mu = \delta_{k_c}, \sigma = \eta_1\right), \quad k \in (1, \ldots, K) \\
\eta_1 &\sim \text{Cauchy}\ \left(\mu = 0, \sigma = \sigma_{\eta_1}\right) \\
\delta_{k_c} &\sim \text{Normal}\ \left(\mu = \delta_c, \sigma = \eta\right), \quad c \in (1, \ldots, C) \\
\eta &\sim \text{Cauchy}\ \left(\mu = 0, \sigma = \sigma_\eta\right) \\
\delta_c &\sim \text{Normal}\left(\mu = -\Delta_{co}, \sigma = \eta_0\right) \\
\eta_0 &\sim \text{Cauchy}\ \left(\mu = 0, \sigma = \sigma_{\eta_0}\right) \\
-\Delta_{co} &\sim \text{Normal}\ \left(\mu = 0, \sigma = \sigma_{\Delta_{co}}\right).
\end{aligned} \tag{A1}$$

The notation largely follows model (3). The extended model (A1) incorporates new parameters: τ_{ykm}, and δ_{m_k}. The τ_{ykm} indicates the site-specific intercept and δ_{m_k} is the mth site-specific "control effect". Each δ_{m_k} is normally distributed around a RCT-specific "control effect" δ_{k_c}, with a standard deviation η_1.
