# Supplementary material for: Developing a Bayesian hierarchical model for a prospective individual patient data meta-analysis with continuous monitoring
Source: BMC Med Res Methodol. 2023 Jan 25;23:25. doi: 10.1186/s12874-022-01813-4 (PMC9875783; doi:10.1186/s12874-022-01813-4)
Supplement: Supplementary file 3 — Additional file 3. Extended model for assessing heterogeneity of treatment effect. [file 12874_2022_1813_MOESM3_ESM.pdf]

Additional file 3 — Extended model for assessing heterogeneity of treatment effect

To explore the impact of a pre-treatment covariate on the CCP effect, we developed another extension to model (3) for investigating the interaction between treatment and a categorical pre-treatment covariate, which S denotes. In this model, an indicator variable d_{kis} that equals 1 if the covariate S for the ith patient in the kth RCT falls in stratum s, and is 0 otherwise.

The model with the interaction between treatment and a pre-specified covariate S is as follows:

$$
\begin{aligned}
\text{logit}\left(P\left(Y_{ki} \geq y\right)\right) &= \tau_{yk} + \boldsymbol{\beta}\boldsymbol{X}_{ki} + A_{ki}\left(\delta_{k_c} + \gamma_{(ks)_c} d_{kis}\right) \\
\tau_{yk} &\sim \text{Normal}\left(\mu = 0, \sigma = \sigma_\tau\right) \\
\boldsymbol{\beta} &\sim \text{Normal}\ \left(\boldsymbol{\mu} = \mathbf{0}, \Sigma = \sigma^2_\beta I_{p \times p}\right) \\
\delta_{k_c} &\sim \text{Normal}\left(\mu = \delta_c, \sigma = \eta\right), \quad c \in (1, \ldots, C) \\
\eta &\sim \text{Cauchy}\ \left(\mu = 0, \sigma = \sigma_\eta\right) \\
\delta_c &\sim \text{Normal}\left(\mu = -\Delta_{co}, \sigma = \eta_0\right) \\
\eta_0 &\sim \text{Cauchy}\ \left(\mu = 0, \sigma = \sigma_{\eta_0}\right) \\
-\Delta_{co} &\sim \text{Normal}\ \left(\mu = 0, \sigma = \sigma_{\Delta_{co}}\right) \\
\gamma_{(ks)_c} &\sim \text{Normal}\left(\mu = \gamma_{cs}, \sigma = \zeta\right), \quad s \in (1, \ldots, S) \\
\zeta &\sim \text{Cauchy}\ \left(\mu = 0, \sigma = \sigma_\zeta\right) \\
\gamma_{cs} &\sim \text{Normal}\left(\mu = -\Gamma_s, \sigma = \zeta_0\right) \\
\zeta_0 &\sim \text{Cauchy}\ \left(\mu = 0, \sigma = \sigma_{\zeta_0}\right) \\
-\Gamma_s &\sim \text{Normal}\ \left(\mu = 0, \sigma = \sigma_{\Gamma_s}\right).
\end{aligned}
\tag{A2}
$$

The extended model includes *RCT-specific* control treatment by covariate interaction parameter $\gamma_{(ks)_c}$. $\gamma_{(ks)_c}$ are assumed to be centered around a control-type mean γ_{cs}. In turn, the γ_{cs}'s are centered around $-\Gamma_s$, the overall "control effect" modification across RCTs.

The pooled effect of CCP (measured by $\log$ OR) across all RCTs for all patients is still Δ_{co}, where as the pooled effect for patients with covariate $S = s$ will be $\Delta_s = \Delta_{co} + \Gamma_s$.
