# Supplementary material for: Developing a Bayesian hierarchical model for a prospective individual patient data meta-analysis with continuous monitoring
Source: BMC Med Res Methodol. 2023 Jan 25;23:25. doi: 10.1186/s12874-022-01813-4 (PMC9875783; doi:10.1186/s12874-022-01813-4)
Supplement: Supplementary file 4 — Additional file 4. Procedure for posterior predictive checks [23]. [file 12874_2022_1813_MOESM4_ESM.pdf]

Additional file 4 — Procedure for posterior predictive checks

The method for calculating Bayesian p-values is as follows. Let D^{original} be the observed dataset and $\boldsymbol{\theta}$ be the vector of parameters. Define D^{rep} as the replicated data that could have been observed. The basic technique is to draw replications of data from the joint posterior predictive distribution $p(D^{\text{rep}}|D^{\text{original}}) = \int p(D^{\text{rep}}|\boldsymbol{\theta})p(\boldsymbol{\theta}|D^{\text{original}})d\boldsymbol{\theta}$ and compare these samples to the actual observed data.[23]

To check whether the co model fits the observed data well, we considered two data-generating mechanisms: the observed data was generated under (i) the proportional cumulative odds assumption and (ii) the non-proportional cumulative odds assumption.

We conducted the following procedure under each data generating mechanism to compute their Bayesian p-values using simulation, respectively:

We fitted the basic co model (model (7)) using the generated data (D^{original}) and estimated the posterior density of a set of parameters $\boldsymbol{\theta} = (\alpha, \boldsymbol{\beta}, \delta_{k_c}, \tau_{yk})$. We sampled 10000 draws from the estimated posterior density of $\boldsymbol{\theta} = (\alpha, \boldsymbol{\beta}, \delta_{kc}, \tau_{yk})$:

$$\boldsymbol{\theta}^1, ..., \boldsymbol{\theta}^{10000} \sim i.i.d \quad p(\boldsymbol{\theta}|D^{\text{original}})$$

Using each draw of $\boldsymbol{\theta}$ and identical explanatory variables $\boldsymbol{X}$ in D^{original}, we generated one hypothetical replicated dataset D^{rep}:

$$D^{\text{rep}\ s} \sim p(D^{\text{rep}}|\theta^s) \quad \text{for} \quad s = 1, ..., 10000$$

Then we had 10000 draws from the posterior predictive distribution $p(D^{\text{rep}}|D^{\text{original}})$.

Because the outcome is ordinal, the test quantities $T(D)$ we used to measure the discrepancy between D^{original} and D^{rep} are the cumulative proportions of subjects that fall into the WHO score categories (i.e., $P(Y \leq y), y = 0, \ldots, 9$).

The estimated Bayesian p-value is the proportion of these 10000 draws for which the test quantities equals or exceeds its realized value:

$$p_B = \frac{1}{10000} \sum_{s=1}^{10000} I(T(D^{\text{rep}\ s}) \geq T(D^{\text{original}}))$$
