# Supplementary material for: Developing a Bayesian hierarchical model for a prospective individual patient data meta-analysis with continuous monitoring
Source: BMC Med Res Methodol. 2023 Jan 25;23:25. doi: 10.1186/s12874-022-01813-4 (PMC9875783; doi:10.1186/s12874-022-01813-4)
Supplement: Supplementary file 5 — Additional file 5. The true value of parameters in the data generation process. [file 12874_2022_1813_MOESM5_ESM.pdf]

746 Additional file 5 — The true value of parameters in the data generation process

Parameter	True value
Δ_{co}	-0.400
δ_1	0.300
δ_2	0.400
δ_3	0.500
$\beta^a_{who_{enroll}=5\|4}$	0.060
$\beta_{who_{enroll}=6\|5}$	0.120
$\beta^b_{age=2\|1}$	0.075
$\beta_{age=3\|1}$	0.150
$\beta^c_{gender=1\|0}$	0.100
$\beta^d_{Symptom\ Days=2\|1}$	0.050
$\beta_{Symptom\ Days=3\|1}$	0.100
$\beta_{Symptom\ Days=4\|1}$	0.150
$\beta_{Symptom\ Days=5\|1}$	0.200
α	0.000

[a] WHO score at baseline

[b] Age: 1 = $<$ 50 years old; 2 = [50,65); 3 = $\geq$ 65 years old

[c] Gender: 0 = female; 1 = male

[d] Duration of symptoms before randomization: 1 = 0-3 days; 2 = 4-6 days; 3 = 7-10 days; 4 = 11-14 days; 5 = 14+ days

Table A2 The true value of parameters in the data generation process.
