# Supplementary material for: Developing a Bayesian hierarchical model for a prospective individual patient data meta-analysis with continuous monitoring
Source: BMC Med Res Methodol. 2023 Jan 25;23:25. doi: 10.1186/s12874-022-01813-4 (PMC9875783; doi:10.1186/s12874-022-01813-4)
Supplement: Supplementary file 6 — Additional file 6. a R code for simulation [33, 44, 45]. b Stan code for the final version of the co model. [file 12874_2022_1813_MOESM6_ESM.pdf]

Additional file 6(a) — R code for simulation

All simulations were performed using R[44], Stan[33], and NYU high-performance computing (HPC) clusters[45]. The code here shows how we simulated the data as in Section Simulation setup - basic model, fitted the final co model, implemented HMC algorithms for Bayesian inference, and computed the posterior medians of the key parameter of interest Δ_{co}. The code for the other simulations is available upon request from the authors.

```
library(simstudy)
library(cmdstanr)
library(data.table)
library(slurmR)
library(posterior)

mco <- cmdstan_model("./primary_co.stan")

#--- generating the study-specific baseline probabilities for each outcome level---#

genBaseProbs <- function(n, base, similarity, digits = 2) {

  n_levels <- length(base)
  x <- gtools::rdirichlet(n, similarity * base)

  #--- ensure that each vector of probabilities sums exactly to 1---#

  x <- round(floor(x*1e8)/1e8, digits) # round the generated probabilities
  xpart <- x[, 1:(n_levels-1)]         # delete the base prob of the final level
  partsum <- apply(xpart, 1, sum)      # add the values of levels 1 to K-1
  x[, n_levels] <- 1 - partsum         # the base prob of the level K = 1 - sum(1:[K-1])

  return(x)
}

nsites <- 9                            # assume 9 RCTs in total

basestudy <- genBaseProbs(n = nsites,
                          base =  c(0.100, 0.107, 0.095, 0.085, 0.090, 0.090, 0.108, 0.100, 0.090, 0.075, 0.060),similarity = 100)

# More detailed information: https://www.rdatagen.net/post/generating-probabilities-for-ordinal-categorical-data/

#--- data definition code ---#

defC <- defDataAdd(varname = "b", formula = 0, variance= .01,
                   dist = "normal")     # RCT specific intercept
defC <- defDataAdd(defC, varname = "size", formula = "75+75*large",
```

```
                    dist = "nonrandom")  # sample size; large=1: 150 patients, large=0: 75 patients
defC2 <- defDataAdd(varname="C_rv",formula="C * control",
                     dist = "nonrandom") # 0=CP, 1= standard of care, 2=non-CP, 3=saline
defC2 <- defDataAdd(defC2,varname="sex", formula = 0.5,
                     dist = "binary")
defC2 <- defDataAdd(defC2,varname="who_enroll", formula = "1/3;1/3;1/3",
                     dist = "categorical")
defC2 <- defDataAdd(defC2,varname="age", formula = "0.25;0.25;0.50",
                     dist = "categorical")
defC2 <- defDataAdd(defC2, varname = "ss",
                     formula = "0.2;0.2;0.2;0.2;0.2", dist = "categorical")
defC2 <- defDataAdd(defC2, varname = "z",
                     formula = "0.05*(ss-1) + 0.1*sex +
                     0.075*(age-1) + 0.06*(who_enroll-1) +
                     (0.3 + b ) * (C_rv==1) +
                     (0.4 + b ) * (C_rv==2) + (0.5 + b ) * (C_rv==3)",
                     dist = "nonrandom")

iter <- function(iternum, defC, defC2, basestudy, nsites,mco)  {

  set_cmdstan_path(path = "/gpfs/share/apps/cmdstan/2.25.0")

  #--- data generation ---#

  dstudy <- genData(nsites, id = "study")               # 9 RCTs
  dstudy <- trtAssign(dstudy, nTrt = 3, grpName = "C") # allocate 3 control conditions
  dstudy <- trtAssign(dstudy, nTrt = 2, strata = "C", grpName = "large", ratio = c(2,1))
  dstudy <- addColumns(defC, dstudy)

  dind <- genCluster(dstudy, "study", numIndsVar = "size", "id")
  dind <- trtAssign(dind, strata="study", grpName = "control")
  dind <- addColumns(defC2,dind)

  setkey(dind, "id")

  dl <- lapply(1:nsites, function(i) {
    b <- basestudy[i,]
    dx <- dind[study == i]
    dx <- genOrdCat(dx, adjVar = "z", b, catVar = "ordY")
    dx[, ordY := factor(ordY, levels = c(1:11))]
    dx[]
  })

  dind <- rbindlist(dl)

```

```
  #--- model estimation ---#

  N = nrow(dind)                                    # number of
    observations
  L <- dind[, length(unique(ordY))]                 # number of levels of
    outcome
  K <- dind[, length(unique(study))]                # number of RCTs
  y <- as.numeric(dind$ordY)                        # individual outcome
  kk <- dind$study                                  # RCT for each
    individual
  ctrl <- dind$control                              # treatment arm for
    individual
  cc <- dind[, .N, keyby = .(study, C)]$C          # specific control arm
     for RCT
  x <- model.matrix(ordY ~ factor(who_enroll) + factor(age) + factor(
    sex) + factor(ss), data =   dind)[, -1]
  D <- ncol(x)
  prior_div <- 8
  prior_Delta_sd <- .354
  eta <- 0.1
  prior_eta_0 <- 0.25
  prior_beta_sd <- 2.5

  studydata <- list(
    N=N, L= L,K=K, y=y, ctrl=ctrl,cc=cc,kk=kk,prior_div=prior_div,
    prior_Delta_sd=prior_Delta_sd,eta=eta,
    prior_eta_0 = prior_eta_0,x=x,D=D,prior_beta_sd=prior_beta_sd)

  fit_co <- mco$sample(
    step_size = 0.1,
    data = studydata,
    chains = 4L,
    parallel_chains = 4L,
    refresh = 500,
    iter_warmup = 500,
    iter_sampling = 2500,
    adapt_delta=0.8
  )

  #--- estimate extraction ---#

  diagnostics_df <- as_draws_df(fit_co$sampler_diagnostics())
  div_num_co <- sum(diagnostics_df[, 'divergent__'])

  res_co <- data.table(fit_co$summary(variables = "negDelta"))[, .(
    median)]
  res_co$div_co <- div_num_co
  data.table(iternum,res_co)
}

#--- Replication ---#
```

```
job <- Slurm_lapply(1:2520,
                    iter,
                    defC=defC,
                    defC2 = defC2,
                    basestudy=basestudy,
                    nsites=nsites,
                    mco = mco,
                    njobs = 90,
                    mc.cores = 4,
                    tmp_path = "/gpfs/scratch/...",
                    job_name = "sim_1",
                    sbatch_opt = list(time = "24:00:00"),
                    plan = "wait",
                    overwrite=TRUE)

site_plasma_all <- Slurm_collect(job)
site_plasma <- rbindlist(site_plasma)

date_stamp <- gsub("-", "", Sys.Date())
dir.create(file.path("/gpfs/home/.../r/", date_stamp), showWarnings =
    FALSE)
save(site_plasma, file = paste0("/gpfs/home/.../r/", date_stamp, "/
    model_co.rda"))
```

Additional file 6(b) — Stan code for the final version of the *co* model

```
data {
  int<lower=0> N;                  // number of observations
  int<lower=2> L;                  // number of WHO categories
  int<lower=1> K;                  // number of RCTs
  int<lower=1,upper=L> y[N];       // vector of categorical outcomes
  int<lower=1,upper=K> kk[N];      // RCT for individual
  int<lower=0,upper=1> ctrl[N];    // treatment or control
  int<lower=1,upper=3> cc[K];      // specific control for RCT
  int<lower=1> D;                  // number of covariates
  row_vector[D] x[N];              // strata indicators  N x D matrix

  real<lower=0> prior_div;         // prior sd of tau
  real<lower=0> prior_Delta_sd;    // prior sd of overall control effect
  real<lower=0> eta;               // prior sd of delta
  real<lower=0> prior_beta_sd;     // prior sd of beta
  real<lower=0> prior_eta_0;       //prior sd of eta_0 (the sd of
    delta_k)
}

parameters {

  real alpha;                      // overall intercept for treatment
  ordered[L-1] tau[K];             // cut-points for cumulative odds
    model (K X [L-1] matrix)
```

```
  real<lower=0>  eta_0;          // sd of delta_k (around delta)


  // non-central parameterization

  vector[K] z_ran_rx;
  vector[3] z_delta;
  vector[D] z_beta;
  real z_Delta;
}

transformed parameters{

  vector[3] delta;               // control-specific effect
  vector[K] delta_k;             // RCT-specific treatment effect
  vector[D] beta;                // covariate estimates
  real Delta;                    // overall control effect
  vector[N] yhat;

  Delta = prior_Delta_sd * z_Delta;
  delta = eta * z_delta + Delta;
  beta = prior_beta_sd * z_beta;

  for (k in 1:K)
    delta_k[k] = eta_0 * z_ran_rx[k] + delta[cc[k]];

  for (i in 1:N)
    yhat[i] = alpha + ctrl[i] * delta_k[kk[i]] + x[i] * beta;
}

model {

  // priors

  alpha ~ normal(0,0.1);
  z_ran_rx ~ std_normal();
  z_delta ~ std_normal();
  z_beta ~ std_normal();
  z_Delta ~ std_normal();

  eta_0 ~ student_t(3,0,prior_eta_0);

  for (k in 1:K)
    for (l in 1:(L-1))
      tau[k, l] ~ student_t(3, 0, prior_div);

  // outcome model

  for (i in 1:N)
    y[i] ~ ordered_logistic(yhat[i], tau[kk[i]]);
}
```

```

generated quantities {

  real OR;                  // overall CCP effect (odds ratio)
  real negDelta;            // overall CCP effect on log scale

  OR = exp(-Delta);
  negDelta=-1*Delta;

}
```
