# Supplementary material for: Developing a Bayesian hierarchical model for a prospective individual patient data meta-analysis with continuous monitoring
Source: BMC Med Res Methodol. 2023 Jan 25;23:25. doi: 10.1186/s12874-022-01813-4 (PMC9875783; doi:10.1186/s12874-022-01813-4)
Supplement: Supplementary file 7 — Additional file 7. Assessing models using a different set of effect sizes. [file 12874_2022_1813_MOESM7_ESM.pdf]

Additional file 7 — Assessing models using a different set of effect sizes

The data were simulated as in Section Simulation setup - basic model but with a set of relatively small effect sizes $(\delta_1, \delta_2, \delta_3) = (0.05, 0.1, 0.15)$.

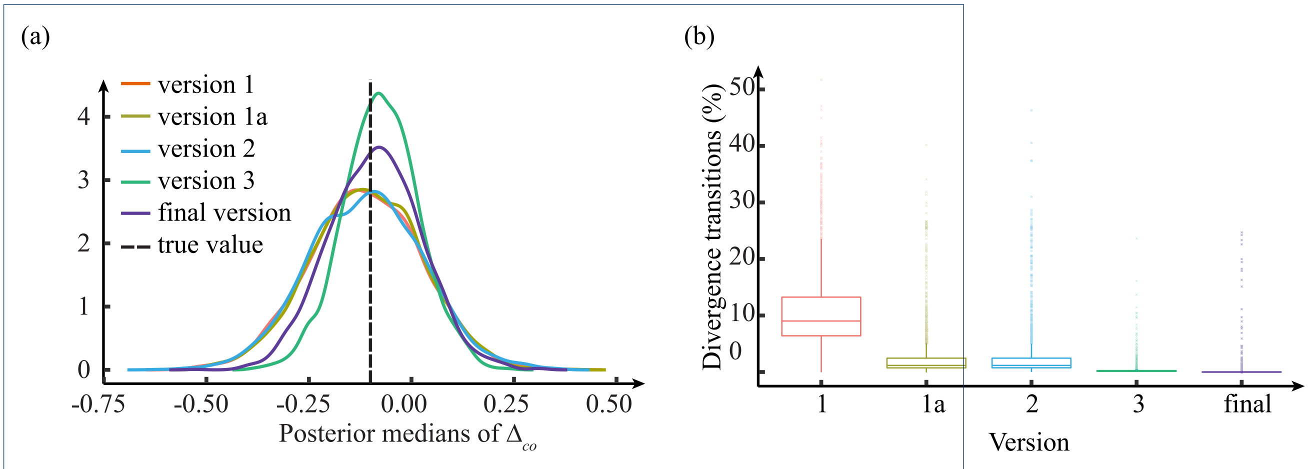

Figure A1 The performance of the five versions of the basic co model: (a) the distribution of posterior medians of the pooled CCP treatment effect Δ_{co} and the true value used to generate the data (true value); (b) boxplots show the median, lower quartile, upper quartile, minimum, and maximum of the number of divergent transitions (%). The proportion of divergent transitions was calculated by (the number of divergent transitions/10,000)$\times 100\%$ in each simulated trial using the five models.

As shown in Figure A1, the results were consistent in this new scenario:

- Version 1: the proportion of divergent transitions was unacceptably high.
- Version 1(a): the divergent transitions decreased relative to version 1, but were not good enough.
- Version 2: the divergent transitions decreased compared to version 1(a), but again were not good enough.
- Version 3: the posterior estimation of Δ_{co} was extracted to the null effect due to the skeptical prior.
- Final version: the true value was almost at the position with the highest probability. The number of divergent transitions was close to or at zero for nearly all simulated trials, indicating that model fitting converged almost every time.
