# Supplementary material for: Developing a Bayesian hierarchical model for a prospective individual patient data meta-analysis with continuous monitoring
Source: BMC Med Res Methodol. 2023 Jan 25;23:25. doi: 10.1186/s12874-022-01813-4 (PMC9875783; doi:10.1186/s12874-022-01813-4)
Supplement: Supplementary file 8 — Additional file 8. The effect of sample sizes on the model’s performance. [file 12874_2022_1813_MOESM8_ESM.pdf]

Additional file 8 — The effect of sample sizes on the model's performance

With the final model in hand, we were able to explore the influence of our assumptions. For example, since the sample size in a prospective meta-analysis could not be predicted, we wanted to know the operating characteristics of the basic co model at different sample sizes. We simulated data as in Section Simulation setup - basic model, which included 900 patients, and two additional scenarios: Scenario (2) that doubles the sample size and Scenario (3) that triples it. For each scenario, we simulated 2500 trials. We compared the accuracy of the estimation.

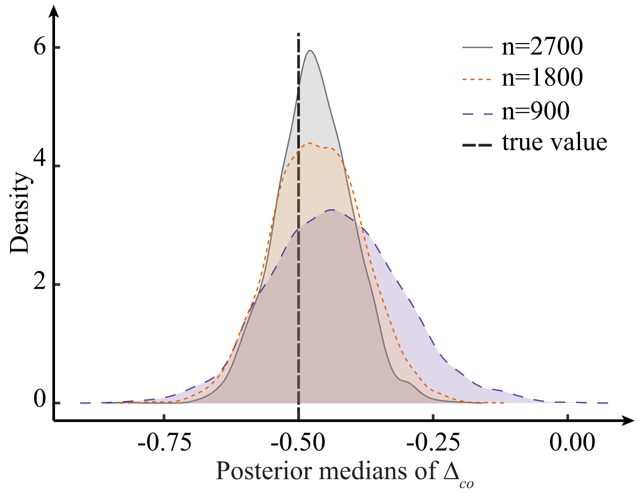

Figure A2 Under different assumptions regarding sample size, distribution of posterior medians (based on 2500 simulated trials) of Δ_{co} and true value used in data generation.

As the sample size increased (see Figure A2), the distribution of posterior medians shifted away from 0 towards the true value. This clearly indicates that a larger sample is required to overcome the postulated highly skeptical prior distribution and demonstrate the effect of CCP treatment.
