# Supplementary material for: Developing a Bayesian hierarchical model for a prospective individual patient data meta-analysis with continuous monitoring
Source: BMC Med Res Methodol. 2023 Jan 25;23:25. doi: 10.1186/s12874-022-01813-4 (PMC9875783; doi:10.1186/s12874-022-01813-4)
Supplement: Supplementary file 9 — Additional file 9. Final model for multi-site RCTs. [file 12874_2022_1813_MOESM9_ESM.pdf]

Additional file 9 — Final model for multi-site RCTs

$$
\begin{aligned}
\text{logit}\left(P\left(Y_{kmi} \geq y\right)\right) &= \alpha + \tau_{ykm} + \boldsymbol{\beta}\boldsymbol{X}_{kmi} + \delta_{m_k} A_{kmi} \\
\alpha &\sim \text{Normal}\ (\mu = 0, \sigma = 0.1) \\
\tau_{ykm} &\sim t_{\text{student}}\,(\text{df} = 3, \mu = 0, \sigma = 10) \\
\boldsymbol{\beta} &\sim \text{Normal}\ \left(\boldsymbol{\mu} = \mathbf{0}, \Sigma = 2.5^2 I_{p\times p}\right) \\
\delta_{m_k} &\sim \text{Normal}\ \left(\mu = \delta_{k_c}, \sigma = 0.1\right) \\
\delta_{k_c} &\sim \text{Normal}\ \left(\mu = \delta_c, \sigma = \eta\right) \\
\eta &\sim t_{\text{student}}\,(\text{df} = 3, \mu = 0, \sigma = 0.25) \\
\delta_c &\sim \text{Normal}\ \left(\mu = -\Delta_{co}, \sigma = 0.1\right) \\
-\Delta_{co} &\sim \text{Normal}\ \left(\mu = 0, \sigma = 0.354\right).
\end{aligned}
\tag{A3}
$$

The parameters in the extended models that were in common with the basic model were set to the prior distributions of the final version in model (7).The extended co model (A3) incorporates new parameters: τ_{ykm}, and δ_{m_k}, which were selected via simulations as those described in Section Extended model for multi-site RCTs. The site-specific intercept τ_{ykm} was set with prior $t_{\text{student}}\,(\text{df} = 3, \mu = 0, \sigma = 10)$. The mth site-specific "control effect" δ_{m_k} was chosen to be normally distributed around a RCT-specific "control effect" δ_{k_c}, with a standard deviation 0.1.
