# Supplementary material for: Developing a Bayesian hierarchical model for a prospective individual patient data meta-analysis with continuous monitoring
Source: BMC Med Res Methodol. 2023 Jan 25;23:25. doi: 10.1186/s12874-022-01813-4 (PMC9875783; doi:10.1186/s12874-022-01813-4)
Supplement: Supplementary file 10 — Additional file 10. Posterior estimations of parameters in the extended model for multi-site RCTs. [file 12874_2022_1813_MOESM10_ESM.pdf]

Additional file 10 — Posterior estimations of parameters in the extended model for multi-site RCTs

Parameter	True value	Model (7)	Model (A3)
Δ_{co}	-0.400	-0.361	-0.362
δ_1	0.300	0.355	0.356
δ_2	0.400	0.371	0.372
δ_3	0.500	0.386	0.386
$\beta^a_{who_{enroll}=5\|4}$	0.060	0.061	0.061
$\beta_{who_{enroll}=6\|5}$	0.120	0.122	0.122
$\beta^b_{age=2\|1}$	0.075	0.076	0.077
$\beta_{age=3\|1}$	0.150	0.158	0.159
$\beta^c_{gender=1\|0}$	0.100	0.107	0.108
$\beta^d_{Symptom\ Days=2\|1}$	0.050	0.051	0.051
$\beta_{Symptom\ Days=3\|1}$	0.100	0.103	0.104
$\beta_{Symptom\ Days=4\|1}$	0.150	0.158	0.159
$\beta_{Symptom\ Days=5\|1}$	0.200	0.208	0.209
α	0.000	-0.002	-0.002

[a] WHO score at baseline

[b] Age: 1 = $<$ 50 years old; 2 = [50,65); 3 = $\geq$ 65 years old

[c] Gender: 0 = female; 1 = male

[d] Duration of symptoms before randomization: 1 = 0-3 days; 2 = 4-6 days; 3 = 7-10 days; 4 = 11-14 days; 5 = 14+ days

Table A3 Mean of estimated posterior medians of each parameter based on 3000 simulated trials.
