# Supplementary material for: Developing a Bayesian hierarchical model for a prospective individual patient data meta-analysis with continuous monitoring
Source: BMC Med Res Methodol. 2023 Jan 25;23:25. doi: 10.1186/s12874-022-01813-4 (PMC9875783; doi:10.1186/s12874-022-01813-4)
Supplement: Supplementary file 11 — Additional file 11. Final model for assessing heterogeneity of treatment effect. [file 12874_2022_1813_MOESM11_ESM.pdf]

Additional file 11 — Final model for assessing heterogeneity of treatment effect

$$\begin{aligned}
\text{logit}\left(P\left(Y_{ki} \geq y\right)\right) &= \alpha + \tau_{yk} + \boldsymbol{\beta}\boldsymbol{X}_{ki} + A_{ki}\left(\delta_{k_c} + \gamma_{(ks)_c} d_{kis}\right) \\
\alpha &\sim \text{Normal}\left(\mu = 0, \sigma = 0.1\right) \\
\tau_{yk} &\sim t_{\text{student}}\left(\text{df} = 3, \mu = 0, \sigma = 8\right) \\
\boldsymbol{\beta} &\sim \text{Normal}\left(\boldsymbol{\mu} = \mathbf{0}, \Sigma = 2.5^2 I_{p\times p}\right) \\
\delta_{k_c} &\sim \text{Normal}\left(\mu = \delta_c, \sigma = \eta\right) \\
\eta &\sim t_{\text{student}}\left(\text{df} = 3, \mu = 0, \sigma = 0.25\right) \\
\delta_c &\sim \text{Normal}\left(\mu = -\Delta, \sigma = 0.1\right) \\
-\Delta &\sim t_{\text{student}}\left(\text{df} = 3, \mu = 0, \sigma = 2.5\right) \\
\gamma_{(ks)_c} &\sim \text{Normal}\left(\mu = \gamma_{cs}, \sigma = 1\right) \\
\gamma_{cs} &\sim \text{Normal}\left(\mu = -\Gamma_s, \sigma = 0.25\right) \\
-\Gamma_s &\sim t_{\text{student}}\left(\text{df} = 3, \mu = 0, \sigma = 1.5\right)
\end{aligned} \tag{A4}$$

The pooled effect of CCP (measured by $\log$ OR) across all RCTs for patients with covariate $S = s$ will be $\Delta_s = \Delta + \Gamma_s$.

The prior distribution of Δ_s is less skeptical than Δ_{co} in model (7) because we are interested in estimating the treatment effect in each stratum of S instead of assessing evidence for overall efficacy. A less skeptical prior spreads out the probability mass, and will not shrink the posterior estimate strongly towards zero; this allows the observed data to have greater influence over the posterior distribution.

The introduction of parameters $-\Delta$ and $-\Gamma_s$ required additional prior distribution assumptions. We found that using t_{student} and $Normal$ prior distributions resulted in similar posterior estimations and divergent transitions. The t_{student} distribution, however, achieved slightly better model convergence and is less restrictive. Therefore, we opted for a t_{student} prior distribution for both $-\Delta$ and $-\Gamma_s$.
