# Supplementary material for: Developing a Bayesian hierarchical model for a prospective individual patient data meta-analysis with continuous monitoring
Source: BMC Med Res Methodol. 2023 Jan 25;23:25. doi: 10.1186/s12874-022-01813-4 (PMC9875783; doi:10.1186/s12874-022-01813-4)
Supplement: Supplementary file 12 — Additional file 12. Final model for the binary co-primary outcome. [file 12874_2022_1813_MOESM12_ESM.pdf]

Additional file 12 — Final model for the binary co-primary outcome

The binary outcome can be properly viewed as a subset of the ordinal outcome. The posterior probability distribution of the OR for the binary outcome will have greater variance than that of the overall cumulative OR, but they effectively measure the same OR. While for efficient use of all available data (i.e., WHO 11-point scale), most statisticians would probably agree that the co model is preferred, the clinicians were reasoning that the most clinically important outcome is the binary measure. This logistic(l) model was included for ease of communication and acceptability by the clinical community.

Assume the event $W = 1$ if the patient has a WHO score $\geq$ 7 (and $W = 0$ otherwise), this outcome is analyzed with logistic (l) regression model as follows:

$$\begin{aligned}
\text{logit}\left(P\left(W_{ki}=1\right)\right) &= \tau_k + \boldsymbol{\beta}\boldsymbol{X}_{ki} + \delta_{k_c} A_{ki} \\
\tau_k &\sim t_{\text{student}}\left(\text{df}=3, \mu=0, \sigma=8\right) \\
\boldsymbol{\beta} &\sim \text{Normal}\ \left(\boldsymbol{\mu}=\mathbf{0}, \Sigma = 2.5^2 I_{p\times p}\right) \\
\delta_{k_c} &\sim \text{Normal}\ \left(\mu=\delta_c, \sigma=\eta\right) \\
\eta &\sim t_{\text{student}}\left(\text{df}=3, \mu=0, \sigma=0.25\right) \\
\delta_c &\sim \text{Normal}\ \left(\mu=-\Delta_l, \sigma=0.1\right) \\
-\Delta_l &\sim \text{Normal}\ \left(\mu=0, \sigma=0.354\right)
\end{aligned} \tag{A5}$$
